# Simultaneous Multiple Liver Metastasis Treated with Pencil Beam Proton Stereotactic Body Radiotherapy (SBRT)

**DOI:** 10.14338/IJPT-20-00085.1

**Published:** 2021-04-22

**Authors:** Neil K. Taunk, Brendan Burgdorf, Lei Dong, Edgar Ben-Josef

**Affiliations:** Department of Radiation Oncology, Perelman School of Medicine, University of Pennsylvania, Philadelphia, PA, USA

**Keywords:** proton therapy, liver metastasis, SBRT, stereotactic body radiotherapy, PBS, pencil beam scanning

## Abstract

Compared with photon stereotactic body radiotherapy (SBRT) plans that may have to use many more penetrating x-ray beams for each isocenter, proton SBRT with ultrahypofractionated doses use fewer beam angles and offer significantly reduced low-dose radiation bath to normal liver tissue. We demonstrate techniques to deliver safe and effective proton SBRT, where planning and organ motion complexity further increased with multiple liver lesions. For treatment planning, we recommend robust and logical beam angles, avoiding devices and encouraging entry perpendicular to the dominant motion, as well as volumetric repainting to mitigate the interplay effect to clinically acceptable levels. This report highlights the significant technical challenges with ultrahypofractionated proton pencil beam scanning liver therapy, how they are managed, and the effectiveness of this treatment.

## Introduction

Ultrahypofractionated proton stereotactic body radiotherapy (SBRT) for liver metastasis is attractive, but technically challenging given organ motion, localization, and worry about the interplay effect. We highlight a case and technical challenges and requisite solutions to treat patients with this technique. For this patient and for our liver proton SBRT program, at simulation we recommend custom whole-body immobilization and a thin abdominal compression belt to reduce intra-abdominal organ motion and avoid bulky stereotactic frames. Challenges to overcome at treatment planning include choosing logical and short beam paths, motion management, and overcoming tumor and normal organ motion during scanning therapy. On-board localization with cone beam computed tomography (CT) is critical for target matching.

Using these methods, we report the case of a patient with oligometastatic cancer with 2 liver lesions. The patient was prescribed pencil beam scanning (PBS) proton liver SBRT with 50 GyRBE in 5 fractions to each lesion, with a generic proton relative biological effectiveness (RBE) of 1.1. The patient has been disease free for 2 years after therapy completion followed with positron emission tomography–computed tomography (PET/CT) and clinical surveillance; no radiation-related sequelae were noted. This therapy can be very attractive for the appropriate patient, and further prospective evaluation is warranted.

## Case Report

**Figure 1.**
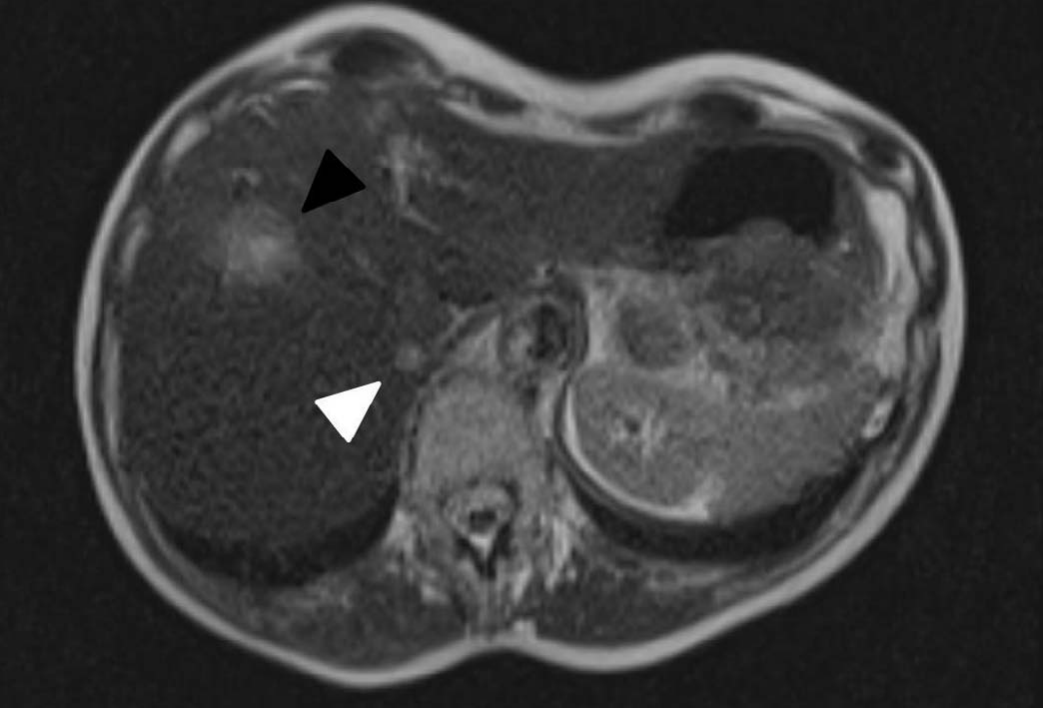
Axial T2 HASTE MRI showing 2.2-cm lesion in segment 8 (black arrow) and 0.8cm lesion in Segment 7 (white arrow).

The patient was a 77-year-old woman diagnosed with right estrogen receptor positive (ER+) breast cancer in 1998, which was treated with mastectomy. In 2010, she experienced right nodal recurrence, which was treated with axillary dissection, postmastectomy radiation (52 Gy to chest and nodes and 46 Gy to supraclavicular nodes), and systemic therapy. In 2018, the patient had recurrence with liver magnetic resonance imaging (MRI) showing a 2.2-cm lesion in segment 8 (biopsy confirmed) and a 0.8-cm lesion in segment 7 (**Figure 1**). PET/CT showed no other disease. The patient started on palbociclib and letrozole. Given oligometastatic disease and prior radiotherapy, patient was offered proton PBS liver SBRT. She had no history of liver disease.

Fiducial markers were placed adjacent to the anterior lesion. The patient was simulated supine, arms up, in full-body custom immobilization using a compression belt for intra-abdominal organ and respiratory motion management with contrast-enhanced 4-dimensional (4D) computed tomography (CT) and MRI simulation. Presimulation fluoroscopic assessment ensured significant reduction in target motion with goal interaction range variations <3 mm for 95% of the target volume [1].

**Figure 2.**
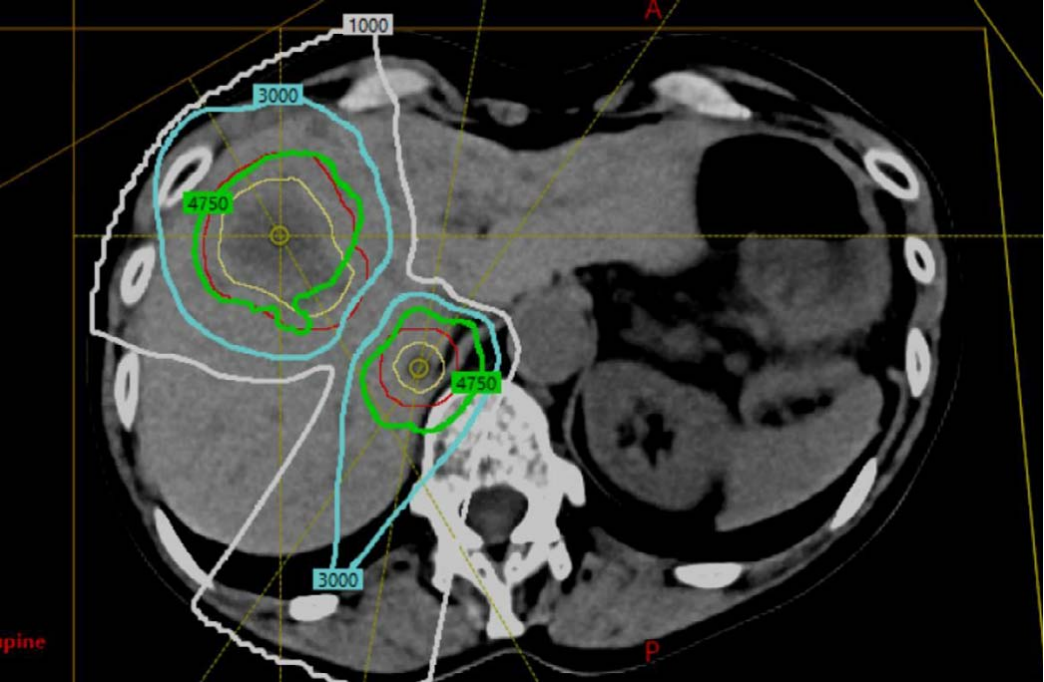
Treatment plan demonstrating 50 Gy in 5 fractions to each lesion and beam pathways (yellow = gross target volume anterior and posterior; red = planning target volume; green = 95% isodose line at 47.5 Gy; blue = 30 Gy isodose line; white = 10 Gy isodose line).

With generic proton RBE of 1.1, the patient was treated with PBS 50 GyRBE in 5 fractions to each lesion, completing therapy September 2018, using a 360° rotational gantry (Ion Beam Applications, Louvain-La-Neuve, Belgium) (**Figure 2**). Key treatment-planning goals are shown in the **Table**. Key metrics were planning target volume 50 with 95% dose > 50 Gy and normal liver gross target volume (GTV) of the primary tumor was V15 Gy < 700 cm^3^. Three right anterolateral oblique beams were used to treat the anterior lesion, and 2 posterior oblique beams were used for the posterior. 4D robust single-field optimization was used with field weighting divided equally among all fields in each plan (each field delivering approximately 1/3 of the dose in the anterior lesion plan and 1/2 of the dose in the posterior lesion plan). Robustness settings used to optimize for internal GTV coverage included 0.5 cm isocenter shifts and 3.5% range uncertainty perturbations. Internal GTV coverage was optimized to ensure 95% of the target volume received at least 47.5 Gy in the worst-case uncertainty dose volume histogram scenario. Given the extent of tumor motion due to respiration, each plan was also optimized for nominal 95% coverage of the PTVs to 47.5 Gy as an additional measure to ensure plan robustness (**Figure 2**). We used methods of 4D dynamic dose calculation to reduce interplay effects via volumetric repainting as previously described [2]. Liver GTV volume was 985 cm^3^. Mean liver GTV dose was 11.6 Gy. Volume of liver GTV receiving ≥15Gy was 284 cm^3^, and the volume receiving <15 Gy was 701 cm^3^. The lesions were treated simultaneously, every other day, using orthogonal imaging and kilovoltage CBCT for alignment. The anterior lesion was aligned to fiducials, and the posterior lesion was aligned to stable vascular and bony anatomy. Mean time on the treatment table was 36.9 minutes and 31.6 minutes for the anterior and posterior lesions, respectively.

**Table.** Dose (D) parameters and goals for proton liver stereotactic body radiotherapy at the University of Pennsylvania.

**Target and critical normal tissue constraints**				
**Structure**	**Dose volume histogram objective,** cGy	**Evaluator**	**Variation acceptable**	**Priority**
PTV_5000	D95%	>5000	3000	2
PTV_5000_Eval	D95%	>5000	3000	2
Spinal cord	D0.03 cm^3^	<3000	3150	1
Spinal cord	D0.1 cm^3^	<2500	2625	1
Spinal cord	D0.35 cm^3^	<2300	2415	1
Spinal cord	D1.2 cm^3^	<1450	1522.5	1
Esophagus	D0.03 cm^3^	<3500	3675	3
Esophagus	D5 cm^3^	<1950	2047.5	3
Heart	D0.03 cm^3^	<3800	3990	3
Heart	D15 cm^3^	<3200	3360	3
Chest wall	D0.03 cm^3^	<4300	4515	3
Chest wall	D30 cm^3^	<3250	3675	3
Skin	D0.03 cm^3^	<3950	4147.5	3
Skin	D10 cm^3^	<3650	3832.5	3
Stomach	D0.03 cm^3^	<3200	3360	1
Stomach	D0.1 cm^3^	<2750	2887.5	1
Stomach	D10 cm^3^	<1800	1890	1
Duodenum	D0.03 cm^3^	<3200	3360	2
Duodenum	D0.1 cm^3^	<3000	3150	2
Duodenum	D5 cm^3^	<1800	1890	2
Duodenum	D10 cm^3^	<1250	1312.5	2
Bowel, small	D0.03 cm^3^	<3500	3675	1
Bowel, small	D5 cm^3^	<1950	2047.5	1
Bowel, large	D0.03 cm^3^	<3800	3990	2
Bowel, large	D20 cm^3^	<2500	2625	2
Gallbladder	D0.1 cm^3^	<4000	4200	3
Lungs	D1500 cm^3^	<1250	1312.5	3
Lungs	D1000 cm^3^	<1350	1417.5	3
Kidneys	D200 cm^3^	<1750	1837.5	3
Liver gross tumor volume	CV1500 cGy (cm^3^)	>700		1

**Abbreviations:** PTV_5000, planning target volume XXXX; Eval, XXXX; CV, XXXX.

**Figure 3.**
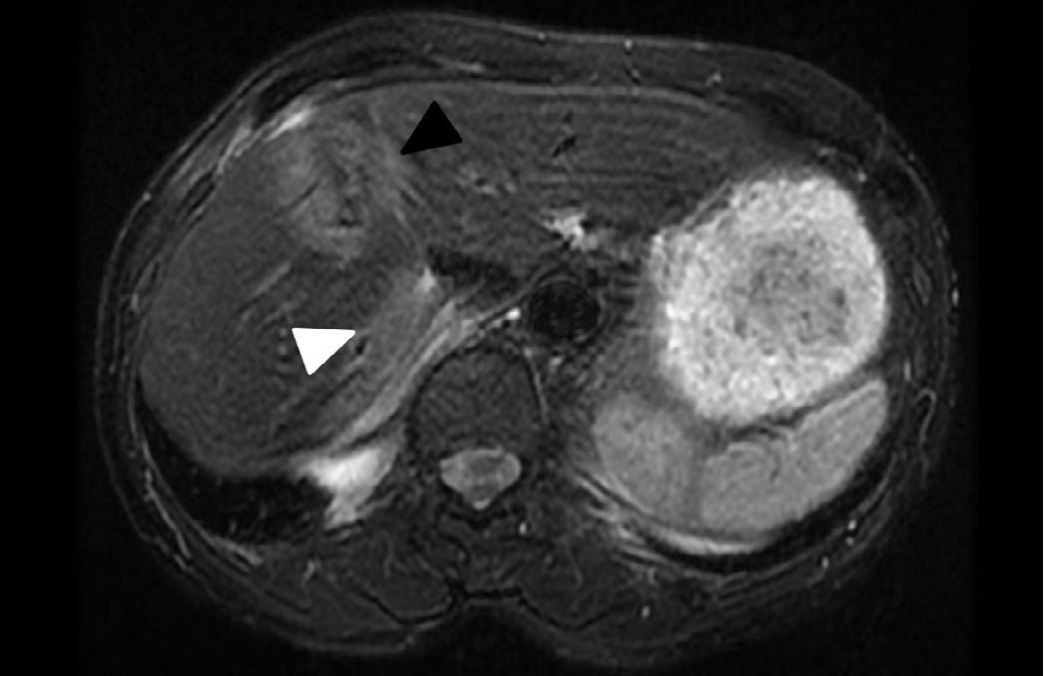
Liver magnetic resonance imaging (axial T2 FRFSE FS POST) at 1.5 years after therapy, which shows stable posttreatment changes at the original anterior tumor location (black arrow). In addition, the proton entry track of the posterior lesion is visible (white arrow). Note an area of normal-appearing liver tissue between the 2 regions with posttreatment changes.

Follow-up PET/CT and liver MRI at 3 months showed complete response to proton SBRT. Subsequent MRIs (**Figure 3**) showed stable proton tracks in liver, most pronounced for the posterior lesion. The patient experienced acute grade 1 fatigue, but no long-term sequelae. Two weeks after SBRT, she began palbociclib and letrozole. She experienced transient elevation of liver enzymes, which resolved after palbociclib discontinuation. The patient resumed letrozole and has been disease free for 2.5 years after treatment followed by PET/CT.

## Discussion

To our knowledge, this is the first published report of simultaneously treated lesions using ultrahypofractionated PBS-proton SBRT. We highlight the value of abdominal compression to mitigate organ motion, 4D CT, repainting, and image-guided therapy to optimize ultrahypofractionated dose delivery in a patient with multiple lesions. Proton SBRT for liver metastasis is attractive. Compared with photon SBRT plans that may use more penetrating x-rays for each isocenter, proton SBRT plans have little exit dose beyond target for each beam, significantly reducing low dose to normal liver.

Proton liver therapy has been evaluated in several prior studies, including PBS proton therapy, although proton ultrahypofractionated SBRT experience is limited, and much long-term clinical data is limited to passively scattered beams [3, 4]. Early proton liver therapy reports demonstrated reduced side effects concordant with reduced liver dose to 700 cm^3^ normal liver, and other organs at risk, particularly dome and central tumors >3 cm [5, 6]. In a phase II study of proton liver SBRT for patients with limited extrahepatic disease and median dose 40 GyRBE in 5 fractions (median biologically effective dose = 72 Gy) using passively scattered beams, local control was 70.1% at 1 year. Treatment was well tolerated, and patients with large liver tumors had excellent control, although the authors indicated that further dose escalation could result in further improved local control. Most PBS liver studies have involved fractionated proton therapy. Dionisi et al [7] reported on 18 patients with hepatobiliary tumors treated with 15 fraction PBS liver therapy with median dose 58 GyRBE. One-year local control was 90%, with excellent toxicity. Kanai et al [8] reported on 20 liver cancer patients comparing 4D robust optimization (4DRO) compared to range-adapted internal target volume , previously treated with gated PBS carbon-therapy, finding 4D robust optimization had higher mean clinical target volume 95% dose using 48 GyRBE in 2 fractions.

However, there are technical challenges to effectively delivering ultrahypofractionated proton liver therapy, such as on-board imaging, motion management, and technology (scattered beams vs PBS) [5]. While respiratory management with deep breath-hold, gating, and compression is well-characterized in photon therapy, it is less well characterized in protons, even though geometric distortion is the predominant driver for poor proton conformality [9–11].

We used rational beam selection and abdominal compression to mitigate organ motion, which we demonstrated to reduce interplay effects levels to that acceptable in clinical practice [1]. Our group has described optimal beam angle selection in PBS liver therapy and found that coplanar right anterolateral and posterior oblique angles are optimal. We used abdominal compression, which we found critical to reduce perpendicular amplitude in moderate (7-10 mm) and significant (>10 mm) motion, although it is likely not necessary in patients with small amplitude motion. Thin, soft, homogeneous compression belts have reduced influence on proton range compared with bulky heterogeneous stereotactic frames. For proton liver SBRT, we recommend robust and logical right anterolateral and posterior oblique angles, avoiding devices. Predominant motion direction (ie, perpendicular to dominant motion) must be reduced, and a compression belt is an attractive solution.

Additional planning techniques are critical to further optimize motion mitigation, including volumetric repainting (using the statistical average of positional errors), spot sequence delivery optimization, and robust optimization. For large lesions PBS produces more conformality than scattered beams, as the scattered spread-out Bragg peak cannot be adjusted for different portions of the target. Motion issues are more pronounced in PBS than scattered beams, given the interplay effect, which is when target motion during active beam scanning and treatment delivery results in differences in the projected doses [12]. The 4D dose must be calculated to evaluate the interplay effect of treatment and repainting, which is beam scanning multiple times during a single fraction, and can be used to reduce uncertainty to clinically acceptable levels. PBS may be more advantageous than scattered beams for additional reasons (eg, scattered beams would require heavy mostly blocked fields producing more neutrons).

Finally, on-board imaging is important for localization and understanding anatomic changes around the target, leading to significant dose perturbation (eg, lung tissue or effusions) [13, 14]. The Imaging and Radiation Oncology Core proton liver phantom demonstrates only 38% pass rate in quality assurance, given that the phantom incorporates multiple lesions and a moving target [15]. The pass rate for all moving phantoms was 63%. CBCT is increasing in proton therapy centers; however, quality of CBCT, pretreatment verification, and robotic repositioning vary. Quality assurance CT provides high-fidelity dose recalculation, but not real-time localization. For proton SBRT, we recommend orthogonal imaging for alignment, if available, and then on-board CBCT matched to liver fiducials, immobile adjacent structures, or liver contour for final localization.

Multiple-lesion SBRT takes the aforementioned technical challenges and then adds liver-sparing challenges and increased time requirements. Even with photons, multiple lesion treatment with photons requires careful treatment planning to ensure adequate liver and organ-at-risk sparing due to intersecting or overlapping beams [16, 17]. We chose optimal beams, as described earlier, to reduce end-of-range uncertainties from coplanar opposed beams, but note that time of delivery was significant given that multiple fields were used to treat each lesion in a single session in a multi-room center.

In summary, to our knowledge, we present the first case of simultaneous treatment of multiple liver metastases treated with PBS-proton SBRT. The patient has remained disease free after proton SBRT for over 2 years on endocrine therapy alone with no chronic toxicity related to radiation. This case represents a technical challenge due to the need for respiratory motion management, mitigating interplay effect, multiple lesion treatment, and use of PBS over scattered beams, but it also highlights an excellent treatment option in proton centers. From the Miami Liver Proton Therapy Conference, there was consensus that radiation therapy is effective for liver tumors and that proton therapy should be considered for select patients with metastasis [18]. There continues to be a need to improve technical barriers to treatment delivery, and additional clinical data are needed considering that outcomes data are largely based on passive scattered beams.
